# A global migration industry in local contexts: Home care and domestic work brokerage in Austria and Sri Lanka

**DOI:** 10.1177/02685809251318642

**Published:** 2025-02-12

**Authors:** Brigitte Aulenbacher, Wasana Handapangoda

**Affiliations:** Johannes Kepler Universitat Linz, Austria; University of Vienna, Austria

**Keywords:** Contextual global sociology, home care and domestic work brokerage, market capitalism and violence, Polanyi, institutional logics, intersectionality, capitalisme de marché et violence, logiques institutionnelles et intersectionnalité, Polanyi, recours à des intermédiaires pour les soins à domicile et le travail domestique, sociologie globale contextuelle, capitalismo de mercado y violencia, intermediación en el cuidado y el trabajo doméstico, lógicas institucionales e interseccionalidad, Polanyi, sociología global contextual

## Abstract

This article investigates the transnational marketization and corporatization of domestic services by brokers. Polanyian, institutional logics, and intersectional perspectives and findings on capitalism and violence provide a theoretical framework to understand brokerage as mode of service provision creating and shaping hybrid and unequal work and care arrangements. The Austrian context is a paradigmatic example for the European mode of senior home care provision by recruiting female migrant workers from Central and Eastern Europe and placing them in Austrian middle- and upper-class households. The Sri Lankan context is a paradigmatic example for a labour brokerage state, which exploits (paid) household labour of women that satisfy the needs of upper- and middle-class households in the Middle East. Contextual global sociology offers a critical lens to compare the ways in which brokerage is politically, socially, and culturally embedded and creates similar and different modes of service provision based on social inequalities.

## Résumé

Cet article étudie la marchandisation transnationale et la corporatisation des services domestiques par des intermédiaires. Les logiques institutionnelles de Polanyi ainsi que les approches intersectionnelles et les recherches sur le capitalisme et la violence fournissent un cadre théorique pour comprendre le recours à des intermédiaires comme un moyen de fournir des services qui crée et détermine des modalités hybrides et inégales de travail et de prise en charge. Le contexte autrichien est un exemple paradigmatique de la modalité européenne de prise en charge des personnes âgées à domicile moyennant le recrutement de travailleuses migrantes d’Europe centrale et orientale et leur placement dans des ménages autrichiens de classe moyenne et supérieure. Le contexte sri-lankais est un exemple paradigmatique d’un État servant d’intermédiaire dans la fourniture de main-d’œuvre, qui exploite le travail domestique (rémunéré) des femmes pour répondre aux besoins des ménages des classes moyennes et supérieures du Moyen-Orient. La sociologie globale contextuelle offre une perspective critique pour comparer les façons dont le recours à ces intermédiaires est ancré politiquement, socialement et culturellement et crée des modalités similaires et différentes de prestation de services basées sur les inégalités sociales.

## Resumen

Este artículo investiga la mercantilización y corporativización transnacional de los servicios domésticos por parte de intermediarios. Las lógicas institucionales de Polanyi, así como las perspectivas interseccionales y los hallazgos sobre el capitalismo y la violencia, proporcionan un marco teórico para comprender la intermediación como un modo de prestación de servicios que crea y configura acuerdos híbridos y desiguales de trabajo y cuidado. El contexto austríaco es un ejemplo paradigmático del modo europeo de prestación de cuidados a domicilio para personas mayores, a través del reclutamiento de trabajadoras migrantes de Europa central y oriental y su colocación en hogares austríacos de clase media y alta. El contexto de Sri Lanka es un ejemplo paradigmático de un Estado de intermediación laboral que explota el trabajo doméstico (remunerado) de las mujeres para satisfacer las necesidades de los hogares de clase media y alta en Oriente Medio. La sociología global contextual ofrece una perspectiva crítica para comparar las formas en que la intermediación está arraigada política, social y culturalmente y crea modos similares y diferentes de prestación de servicios basados en las desigualdades sociales.

Since the 1990s, in the second era of globalization and under the auspices of neoliberalism, the brokerage of domestic services has become a flourishing industry. While domestic service brokerage is a global business, we use domestic service as an over-arching term for domestic work and home care. Depending on the context, it covers cleaning, cooking, caring (be it child or senior care), and some more everyday life assistance. Brokerage agencies are powerful actors, shaping the business and influencing the working conditions. Our article aims at shedding light on home care and domestic work by investigating the Austrian and Sri Lankan contexts that offer timely relevant empirical examples in exploring the role of domestic service brokerage in contemporary market capitalism. The article refers to Polanyian concepts, historical analysis of capitalism and violence, neo-institutionalism, and intersectional theory to understand the ways in which the industry of brokerage works (1). After a description of the methods (2), we analyse the Austrian and Sri Lankan contexts of domestic service brokerage (3). Finally, our comparison and conclusion reflect on the brokerage industry within local contexts (4).

## Theorizing the embeddedness of domestic service brokerage in market capitalism

In Austria and Sri Lanka, as well as in other countries, brokerage agencies are intermediators offering domestic services by recruiting migrant workers, mostly female, and placing them as live-ins^1^ in private households. Instead of the historically widely spread informal and individual commodification of domestic work and home care, brokerage is a marketized and corporatized mode of service provision (Farris and Marchetti, 2017). Hence, combining different theoretical perspectives, we analyse the embeddedness of this industry in neoliberal market capitalism.

Starting from a Polanyian perspective, as a core element of domestic service brokerage, we can identify the transformation of the ‘human activities’ of labour and care into ‘fictitious commodities’ (Polanyi, 2001: 75). Polanyi distinguishes ‘genuine’ from ‘fictitious commodities’: the latter cover elements – in his view: labour, nature, and money, and we add: care (Aulenbacher and Leiblfinger, 2019) – which have neither been ‘produced for sale’ nor, with regard to labour and care, can ‘be detached from the rest of life, be stored or mobilized’ (Polanyi, 2001: 75). In his view, in the capitalist ‘market society’ – driven by the ‘commodity fiction’ of economic liberalism (Polanyi, 2001: 75) and giving priority to the economic principle of ‘market exchange’ to organize economy and society (Polanyi, 2001: 45ff.) – ‘fictitious commodities’ are dealt with like ‘genuine commodities’. Nevertheless, unlike ‘genuine commodities’, subordinating the commodification of labour and care solely to supply and demand threatens their substance and puts livelihood at risk (Polanyi, 2001: 71ff.).

Regarding the availability of labour and care as ‘fictitious commodities’, female migrant domestic and care workers can be considered as an exchangeable workforce dealt within competitive trans- and international labour markets. However, the brokerage industry depends on the demand of households as potential clients. In terms of the best fit for individual households, brokerage agencies strive to mediate not only the available, but also the most appropriate workforce and thus try to deliver or even guarantee services of promised quality. In case of live-ins, this often means that workers must subordinate their own life to expectations of individual households (Aulenbacher and Leiblfinger, 2019; Lutz and Benazha, 2024; Parreñas, 2021).

Under the auspices of neoliberalism, brokerage of domestic services seems to follow the ‘utopian principle of a self-regulating market’ (Polanyi, 2001: 157), or – as agencies paraphrase – the ‘free market’. In this view, labour as well as consumer markets do not seem to be embedded in society. But in the case of the workforce, the ideology of the free market and the related imagery of equal contract partners ignores that workers often are forced to migrate for economic or political reasons and are available under conditions they cannot be negotiated. In the case of private households, the ideology of the free market and contract addresses those who can afford to pay for domestic services (middle and upper classes) but neglects to take into account the asymmetry between them and the workers (Aulenbacher et al., 2020a; Wee et al., 2020). Drawing on classics like Marx, Weber, and Polanyi, Gerstenberger’s (2018) global historical reconstruction of the relation of the market and violence (p. 15) discusses the capitalist free work contract as ideology and part of what Galtung names ‘structural violence’, which is understood as systemic violence inherent to the capitalist economy. Although it is an impersonal violence, it produces vulnerabilities, precarities, and dependencies by making economic use of social inequalities in the relations of gender, race, and class^2^ (Abraham 2021, Abraham and Vasil, 2024).

From a Polanyian perspective, we analyse (neo)liberal market exchange as something disembedded and embedded in society. Brokerage itself can be understood as a result of a historical ‘movement’ (Polanyi, 2001: 71ff.) towards neoliberal ‘marketization’ and ‘corporatization’ of domestic services (Farris and Marchetti, 2017). The creation of the respective labour and consumer markets has been supported by different actors and institutions, politically shaped and regulated, although they are considered ‘self-regulating’ (Polanyi, 2001: 138f) or free markets. Regarding violence, Gerstenberger’s (2018) investigation shows that such elements like state intervention, and legislation have been identified to be critical for reducing ‘direct violence’ (e.g. interpersonal violence, acts of caprice) in the history of capitalism. Notwithstanding their taming influences, her study also shows how ‘direct’ or – as we refer to Williams (2005) – ‘personal’ and ‘structural violence’ interfere in the case of market exchange and delimited exploitation (*entgrenzte Ausbeutung*) of labour. Furthermore, violence can be co-created and co-constituted by political intervention and regulation of market capitalism (Gerstenberger, 2018: 18). This aspect is of relevance for our research field. On the one hand, brokerage agencies are powerful actors, as marketeers promoting their business and as stakeholders striving to politically influence their business conditions. On the other hand, they depend on the regulation of the market and their business in the respective care, migration, employment and gender regimes of the societies involved in the transnational arrangement and make use of them (Aulenbacher et al., 2020b; Leiber and Österle, 2022). In both perspectives, brokerage agencies act as ‘bureaucratic interpreters’ (Wee et al., 2020) of the administrative and political rules for all parties involved.

To investigate this embeddedness of domestic service brokerage and the creation of respective domestic work and home care arrangements, we refer to the neoinstitutionalist ‘institutional logics’ perspective (Thornton et al., 2012) and intersectional theory (Crenshaw, 1989). Both help to better understand how the marketization and corporatization of domestic services are related to the institutional and normative order of the respective societies (Thornton et al., 2012: 65ff.), the division of labour and the relations of gender, race/ethnicity, and class inherent to it (Fraser, 2022; Kofman and Raghuram, 2015).

From the ‘institutional logics’ perspective, the logics of the community, family, state, religion, profession, market, and the corporation are sensemaking orientations rooting in the institutional and normative order of societies and giving identity, authority, and legitimacy to individuals and organizations and their activities (Dammayr, 2019: 51ff.; Thornton et al., 2012: 3ff.; Ocasio et al., 2017). Individuals – for example, the domestic and care workers, employers, care recipients and relatives in the household, staff members of agencies – as well as organizations – for example, brokerage agencies, government offices, stakeholders’ organizations – refer to them in their everyday practice. In this perspective, domestic service brokerage is influenced by different orientations, which can conflict with, influence, or transform each other (Aulenbacher et al., 2020a). Intersectionality is an analytical framework for understanding the ways in which inequalities based on gender, class, race, and other forms of discrimination interact and intersect to shape the division of labour and to produce unique combinations of everyday experience (Crenshaw, 1989) influencing individual and organizational activities. Intersectionality studies show how social inequalities in the relations of gender, race, and class are reproduced through contemporary global capitalism (Bohrer, 2018; Fraser, 2022; Kofman and Raghuram, 2015) and are fundamental to the marketization and corporatization of domestic services (Farris and Marchetti, 2017; Marchetti, 2022; Parreñas, 2021). As neither labour nor care can be ‘detached’ (Polanyi, 2001) from the individual workforce, the brokerage industry inevitably involves a range of orientations and practices that affect recruitment strategies as well as images of the appropriate workforce and division of labour (Aulenbacher et al., 2020a; Findlay et al., 2013). Connecting demand and supply forces, brokerage agencies act as ‘career counsellors’ (Handapangoda, 2024) by promising to organize the best fit between the private household’s expectations and the worker’s profile and as ‘language brokers’ (Dorner et al., 2007) prescribing the anticipated live-in arrangement and composition of paid and unpaid labour.

Through the optics of institutional logics and intersectionality, domestic service brokerage can be analysed as embedded in the institutional and normative order of the respective societies giving shape to the domestic work and home care arrangements. These arrangements are hybrid in the sense that they refer to and amalgamate the logics of the market, corporation, profession, state, family, community, religion, and so on, that all parties involved refer to and create new divisions and combinations of paid and unpaid labour. And they are unequal arrangements, stratified in the relations of gender, race, and class, and structurally and (potentially) personally violent and exploitative. Both – the hybridity and inequality of the live-in domestic work and home care arrangements – are strongly interwoven (Aulenbacher et al., 2020a). Just to give one example: the logics of the market – demand and supply – can be influenced by all other logics; for example, for households may the religion, professionality, ethnicity, and so on, of the workers play a role; this can make a difference in the division of labour in the household, the prices for, and costs of the workforce and the services sold and bought (Cheng, 2013; Prieler, 2021).

Last but not least, the ‘institutional logics’ perspective let us take into account that such orientations are also remarkable on the field level (Thornton and Ocasio, 1999). Brokerage may be contested by the parties involved – by labour disputes, care struggles, and so on (Marchetti, 2022; Marchetti et al., 2021) – but nevertheless the industry gains identity, authority, and legitimacy in the field of domestic work and home care. It creates and shapes new modes and forms of domestic service provision and respective work and care arrangements. Thereby and more or less in line with the regulation by the state, its politics, policies, legislation, the brokerage industry has become an established part of the capitalist economy (Aulenbacher et al., 2024; Leiber and Österle, 2022; Marchetti, 2022; Parreñas, 2021). Coming back to our entry point, the marketization and corporatization of the ‘fictitious commodities’ (Polanyi, 2001: 75) labour and care by brokerage agencies are part of a global migration business but take shape depending on their local political, social, and cultural embeddedness in the institutional and normative order of the societies involved. Regarding the profile of domestic work and home care brokerage on the field level, this means that we find different modes and forms of domestic service provision producing different live-in work and care arrangements in different regions of the world.

As Abraham (2019) – developing her ‘contextual global sociology’ approach – argues, while the society in the twenty-first century continues to evolve, with both old and new issues and dilemmas that are often global, they require understanding within specific contexts. Correspondingly, the Austrian and Sri Lankan case of domestic service brokerage represents the making and shaping of a global migration industry in the most diverse local contexts. Therefore, their investigation promises insight into this business model and the respective hybrid and unequal modes and forms of domestic service provision and work and care arrangements.

## Research methods

The case studies draw on two projects and use qualitative methods because they are best suited to provide rich, detailed, and context-specific insight into the complexity of migration brokerage. The methods of the Austrian country study (conducted 2017–2021) include policy, regime, and media analyses; expert interviews with all relevant stakeholders; website analyses of agencies and expert interviews with agencies’ owners and executive staff; group discussions with carers and relatives of care recipients; case studies involving non-participant and participant observations; episodic interviews with representatives of brokerage agencies, carers, clients, and relatives.^3^ The methods of the Sri Lankan country study include qualitative fieldwork in Sri Lanka, Saudi Arabia, and Kuwait (2020–2022). This involved direct personal and digital interviews based on episodic inquiry.^4^ While brokerage agencies served as the focal point of research in both contexts – the Austrian and Sri Lankan – other participant groups, as integral components of the brokerage business, offered critical, context-specific perspectives on brokerage, thus puzzling out the role and engagement of a highly contested yet crucial player in the global migration business.

Data analysis was carried out using episodic, thematic, and narrative inquiry. In the former, the episodes were compared and the narratives were synthesized and integrated into ‘a chain of relevant situations’ (Flick, 2000: 81; cited in Mueller (2019)), while thematic inquiry involved identifying and examining common themes, patterns, or regularities across the data. The latter – narrative inquiry – entailed systematically interpreting how participants gave meaning to their daily lives through narratives. Data comparison was organized in terms of the main topics of the interviews. The two case studies allow in-depth, multifaceted, and nuanced explorations of home care and domestic work brokerage in their real-life settings, which can only be understood in contextual ways (Abraham and Tastsoglou, 2016; Crowe et al., 2011). Therefore, from the perspective of ‘contextual global sociology’ (Abraham, 2019), our article is an attempt, inter alia, to capture and reflect on the ways in which brokerage is influenced and shaped by global and local contexts in which it takes place.

## Domestic service brokerage in Austria and Sri Lanka

The term ‘domestic services’ covers a wide range of services offered to private households by brokerage agencies. However, there are significant differences between the Austrian and Sri Lankan contexts that we need to consider before we go into the details.

Although live-in work is historically well known in Europe, the Austrian case represents the making of a flourishing new branch in the migration industry that dates to the 1990s. This business is highly specialized on the intermediation of senior home care and is practised in many European countries in face of demographic change, declining public services, and changing intergenerational family and gender arrangements and the respective care gaps. It is based on the availability of migrant workers in and out of Europe who can be placed as live-ins, providing householding, companion, everyday life assistance, medical services, and transforms the formerly informal live-in work into a formalized home care provision (Aulenbacher et al., 2024; Leiber et al., 2021; Leiber and Österle, 2022). While in some parts of Europe long distance and long-term migration play a role, the most significant form of mobility takes the form of circular migration between East, Central, South, and West European countries. The new European Union (EU) member states serve as sending – and some also as receiving – countries in the inner European economic and wealth gap where poverty and unemployment are major push factors (Aulenbacher et al., 2024; Bahna and Sekulová, 2019; Melegh, 2023). In the Austrian context, recruitment of care workers from Slovakia and Romania is most significant but also Hungary and Croatia have become remarkable sending countries (Leiblfinger and Prieler, 2018).

The Sri Lankan context represents the global model of domestic work brokerage and in particular long distance and long-term South to South migration, which dates to the early 1980s. The oil-rich Gulf represents the key destination region for Sri Lankan women who migrate as live-in domestic workers. For decades, following Sri Lanka’s economic liberalization reforms in 1977, women from backward socioeconomic locations have filled the reproductive labour gaps in affluent private households in the Gulf. On the one hand, their journeys of migration explain the extreme human conditions, such as poverty and unemployment, upon which women enter the transnational labour market (Handapangoda, 2023; Withers, 2019). On the other hand, they speak of the pull factors in the Gulf, that is, the changing sociocultural and lifestyle factors followed by the 1973-Arabian Oil Boom and recently, women’s growing workforce participation, for example, in Saudi Arabia caused by the Saudization Program in 2003, encouraging more and more Saudi women to enter the labour market as waged workers and thereby creating a growing demand for paid migrant domestic work (Handapangoda, 2023). Most Sri Lankan women’s migration journeys are facilitated by the support structures offered by private brokerage agencies. The brokerage business thus constitutes an indispensable node of Sri Lanka’s care migration industry that offers a lifeline to millions of Sri Lankan women and their significant others who stay behind.

### Senior home care brokerage in Austria

In Europe, Austria’s senior home care brokerage figures as neoliberal forerunner model. In the conservative welfare state, the logics of the family, state, profession, and market converge (Shire, 2015): ‘explicit familialism’ (*expliziter Familialismus*) is a leading orientation in the Austrian welfare state taking the family – and implicitly female relatives – and less the public sector into consideration to provide senior home care (Leitner, 2013). In this setting, the state’s cash-for-care policies (uncommitted care allowance, additional federal allowance), legalization of live-in work, and its professional acceptance as personal care by the Home Care Act (2007) as well as the separation between personal care and its brokerage as free trades in their own right (2015) converge into an increasing marketization and corporatization of senior home care with a neoliberal self-employment model at the core. More than 1000 brokerage agencies – sole proprietors, enterprises in family ownership, transnational companies, agencies related to NPOs, and so on – place 60,000 self-employed care workers, mostly women at the age of 40–60 years, in 32,000 private households (Aulenbacher et al., 2020b; Leiber and Österle, 2022; Leiblfinger and Prieler, 2018).

In the neoliberal self-employment model, brokerage agencies deal with the ‘fictitious commodity’ care by providing householding, companion, everyday life assistance, and, under special conditions, medical services. Their clients – in the brokerage agencies’ understanding: primarily the care recipients and/or their relatives – can chose between packages including administrative and care services but also conflict mediation, the exchange of the care workers or visits in the households to control the quality, and so on. The households must pay for the brokerage agency’s fees as well as the honorarium of the care workers (average costs: 2500€–3000€/month). And brokerage agencies deal with the ‘fictitious commodity’ labour by evaluating the demands of the household, searching for the appropriate workforce promising to fulfil the respective expectations and to be the best fit for the care recipients’ needs, accompanying the care workers to the household and, often, organizing their transport and/or administrative belongings as self-employees concerning social insurance, registration of the business, and so on. The care workers’ honorarium is calculated in the frame of the service provision (average honorarium of the care workers: 60€–90€/24 hours) (Aulenbacher et al., 2020b; Leiber and Österle, 2022). On the field level, brokerage combines the ideal of family care with a marketized and corporatized mode of senior home care provision supported and co-regulated by the welfare state.

Brokerage agencies give shape to the arrangement by their knowledge and practices. They act as ‘bureaucratic interpreters’ (Wee et al., 2020: 994) drafting the contracts for all parties involved (contract triangle: household-brokerage agency, worker-brokerage agency, household-worker), sometimes making use of model contracts provided by the Chamber of Commerce which represents brokerage agencies as well as care workers (mandatory membership). As the established Austrian self-employment model is not covered by the labour law, working conditions are neither regulated nor subject of collective bargaining. They are indirectly and directly influenced by the brokerage agencies’ offered services but must be negotiated individually by the care workers and the care recipients and/or their relatives. As ‘career counsellors’ (Handapangoda, 2024) and ‘language brokers’ (Dorner et al., 2007) brokerage agencies influence the live-in care arrangement by predominantly communicating the demands and expectations of the care recipients and their relatives and the duties of the care workers who are addressed to perform their services as self-employees in the given order of the household. In the Austrian context, the private household is a difficult workplace and living space because of the blurring boundaries between work, leisure time, and intimacy which promote delimited exploitation (Gerstenberger, 2018) in terms of overwhelming demands, working hours, and times of availability and the contestation of breaks (informal standard in the field: 2 hours per 24 hours). Furthermore, if care workers live alone with multi-morbid care recipients, they often cannot leave the household (Aulenbacher and Leiblfinger, 2019; Aulenbacher and Prieler, 2024).

According to the findings of our project, the working conditions in the household are often scandalized as ‘*modern slavery*’ (care workers, stakeholders) in face of the inherent structural violence related to expectations like 24-hour availability of the care workers at cost of their self-care but also moments of personal violence like insufficient board and lodging. In terms of intersectionality, the live-in care arrangement in Austria is based on the availability of a cheap female workforce from abroad – care workers who are forced to accept poor working conditions and cannot afford to live out of the household of the care recipients for financial reasons.

In accordance with the government’s social policies, brokerage agencies are protagonists of the neoliberal self-employment model, from their standpoint, guaranteeing legal and affordable domestic services. Although convinced marketeers in a Polanyian sense, leading agencies, nevertheless, criticize the competition in the market: ‘*(. . .) agencies offering more quality (. . .) are closer together (. . .), but there are many agencies (. . .) simply intermediating the ladies (care workers*, authors*) by, indeed, price dumping*’ (brokerage agency). From this perspective, price dumping strategies often are interrelated with the search for cheaper and more exploitable workforce in the poorest regions of Eastern Europe. However, recruitment strategies at all are framed and guided by ethnic stereotypes (Prieler, 2021). Just to give one example: while Romanian carers are considered to accept poor working conditions because of their ‘*mentality*’, Slovakian and Hungarian care workers are preferred because *‘they take responsibility (. . .) and therefore can be qualified to work in the frame of the self-employment model*’ (brokerage agency). However, imageries of the appropriate care worker are not only ethnicized but also feminized and refer to different logics; one of them is the family logic. Advertised as quasi-family members (like daughters, granddaughters) doing ‘love-work’ (Lutz, 2002: 90), female care workers seem neither professional nor doing hard work although both are part of the brokered domestic services (Aulenbacher et al., 2020a).

However, leading brokerage agencies also criticize the poor working conditions because they remarkably contribute to the bad image of the migration industry. They address the government to realize stronger financial support of the welfare state. Furthermore, they plea for support for the migration industry’s self-regulation by and self-commitment to shared minimum standards (as, for example, covered by the Austrian quality seal ÖQZ-24 brokerage agencies can apply for) (Aulenbacher et al., 2020b; Aulenbacher and Prieler, 2024; Leiber and Österle, 2022). The idea behind is a shift from price- to quality-based competition on the consumer market supported by the state: ‘*(. . .) if the clients receive additional money and can be sure that they will receive quality, then they will decide for quality*’ (brokerage agency) instead of low-price offers of competing agencies undermining the established professional standards by the high-price agencies. Such efforts to improve the services shall and can but must not affect the working conditions in the frame of the neoliberal idea of the free work contract and will of the parties involved which are at the core self-employment model.

The ideology of free contracts of equal partners is also of interest regarding the interference of structural and personal violence in the household. Regarding sexual harassment, care workers describe the work as ‘*dangerous*’ if their room is not able to be locked and secured. Brokerage agencies know this: ‘*This is more from the female perspective, because most of them (care workers*, authors*) already have some bad experiences (. . .) because of dementia or aggression in quotation marks to the point that one will be touched bodily (. . .). However, one person interprets it, whether it is really caused by illness or harassment, everything is possible, and, of course, the ladies (care workers*, authors*) are cautious and say: “I am living alone with him in the house, (. . .) I don’t feel good there”’.* (brokerage agency) However, brokerage agencies understand such experiences as the informal side of the live-in work and care arrangement and not as inherent to it in face of the household as difficult workplace and the social inequalities making live-out impossible.

On the field level, by marketizing and corporatizing domestic services, senior home care brokerage shapes hybrid and unequal modes of care provision and work and care arrangements. Supported by the state, they promise to provide professional services and replace family by migrant care for Austrian middle and upper classes by recruiting exploitable live-in workers – what on the flipside leads to care drain from Central and Eastern European households and transnational care gaps. Furthermore, brokerage agencies explicitly address the Austrian welfare state to interfere by (market) regulation and ask for (more financial) support to improve their services in favour of the clients’ households without affecting the self-employment model. In face of social inequalities inherent to migration brokerage and following the ideology of the free contract and will of equal partners on free markets, this model – uncovered by the labour law – does not protect from poor or violent working conditions in case of the given vulnerable workforce.

### Domestic work brokerage in Sri Lanka

For decades, Sri Lanka successfully trades in ‘love-work’ (Lutz, 2002: 90) at a transnational level, turning itself into a ‘labour brokerage state’ (Rodriguez, 2008: 794), producing, distributing, and regulating migrant domestic workers, whose remittances put food on the table. Currently, women contribute to nearly 35% of Sri Lanka’s total labour outflow, of which a vast majority –75%– are employed as live-in domestic workers in the oil-rich Arabian Gulf, which receives more than 95% of Sri Lanka’s domestic labour exports (Sri Lanka Bureau of Foreign Employment (SLBFE), 2020). Brokerage agencies play an essential role in Sri Lankan women’s aspirations for mobility in a dis/embedded brokerage market. From the lens of ‘conditionality’ (Goldring and Landolt, 2013: 4), their migration trajectories, experiences, and circumstances are significantly conditional upon the intermediation of brokerage agencies. Having no other means of support, little or no administrative literacy, migrant domestic workers excessively depend on brokerage agencies in steering the Arabian Gulf migration and border bureaucracies. Every three out of four migrant domestic workers in Sri Lanka depend on the support structures offered by brokerage agencies for their journeys abroad^5^ (SLBFE, 2021).

The recruitment of migrant domestic workers takes the form of a transnational operation in and between Sri Lanka and the countries of destination in the Arabian Gulf, engaging both public and private, local and foreign, and formal and informal actors. Most of the migration business is performed by licenced brokerage agencies in Sri Lanka in partnership with brokerage agencies, licenced, in destination countries. The contractual partnership is established based on a Memorandum of Understanding (MoU) called the *musaned* system. Therefore, in a state and regulated brokerage market, brokerage agencies organize and facilitate the recruitment of migrant domestic workers across borders in many ways. They sell services including but not limited to finding employers, matching between employers and domestic workers, fulfilling recruitment formalities and destination-specific logistics, completing information needs and follow up.

Importantly, the recruitment of migrant domestic workers is based on certain criteria, for example, gender, age, salary, experience, training, language skills (Arabic), religion (Muslim/non-Muslim), civil status, and physical appearance and skin colour (race). Combining the logics of the profession, family, market, and the state, these standards are set by both employers and the state. They are negotiable; brokerage agents negotiate the standards, both formally and informally, thus actively fashioning knowledge and orientations between the state, employers, and migrant domestic workers. Capitalist recruitment processes are therefore mediated and shaped by the norms, values, beliefs, preferences, and expectations of the appropriate workforce, which is case-specific; the specifications vary from one employer/private household to another. As an agent comments: ‘It’s our duty to give the right person for the right job’. Brokerage agents therefore exercise a greater degree of agency in determining the extent of separation between market and non-market institutions, thereby shifting between self-regulation of the market, at one end, and absence of the market, at the other. They negotiate and regulate the desirability of migrant domestic workers in terms of bodily forms (e.g. healthy, not too old or too young) (Findlay et al., 2013) and bodily expressions and norms (e.g. obedient, hardworking), thereby determining ‘who gets what’ in a transnational brokerage market that constructs and (de-)valorizes migrant bodies along physical, behavioural, and cultural traits: ‘60% or 70% [employers] prefer migrant domestic workers below 40 years. (. . .) fair or light-skinned because some employers don’t like dark-skinned women. (. . .)’ (two agents). Brokerage agents thus produce hybrid and unequal domestic work arrangements and the image of the ‘ideal’ migrant domestic worker by combining and negotiating different logics (Liang, 2011).

Agents produce the employability and marketability of migrant domestic workers. They act as ‘language brokers’ (Dorner et al., 2007: 458), translating and interpreting between employers, the state and migrant domestic workers, and vice versa; they render them legible and meaningful to one another (Chávez, 2009; Wee et al., 2020), not simply in the sense of linguistic exchange, but also as a social intervention. To this end, brokerage agents exploit the language as well as the linguistics. For example, by calling migrant domestic workers *mage lamaie* (‘my girls’) and female Arab employers ‘madam’, they activate a process of meaning-making, through which power inequalities are systematically and consistently formulated. They construct and portray migrant domestic workers as easily controllable and subdued as opposed to employers who are portrayed as powerful figures who rightfully deserve respect and obedience on the part of migrant domestic workers. Brokerage agents are ‘career counsellors’ (Handapangoda, 2024: 257). They police and guide migrant domestic workers through information in the forms of advice, assurances, explanations of possibilities and warnings in performing the duties of the job and fulfilling the often exhausting demands in the private Arab household (Handapangoda, 2024). This training from below fills a crucial information or knowledge gap of migrant domestic workers and, at the same time, shapes and transforms them into desirable and marketable migrant bodies: diligent, docile, and dutiful. While there is no cause–effect relationship between brokerage agents and migrant domestic workers, the former remain crucial players during the entire migration process: from recruitment and placement of migrant domestic workers to their work and stay in the Arabian Gulf, for example, as conflict mediators and exit options in the case of employer’s exploitation, to return and repeated migration, where migrant domestic workers often rely on agents for migration.

Brokerage agencies are ‘bureaucratic interpreters’ (Wee et al., 2020: 994). Dealing with the ‘fictitious commodity’ labour, they translate policy texts into tacit and explicit advice and put them into practice through acts of drawing up contracts and offering advice to potential migrant domestic workers and employers (Wee et al., 2020). In this way, agencies assist migrant domestic workers to navigate and, at times, subvert migration and border controls, which are highly bureaucratic and not necessarily migrant-friendly. They often operate outside the regulatory umbrella of the state and undermine legal/illegal divide in socially constructed state/market boundaries (Alpes, 2017), colluding with various actors, including migrant domestic workers themselves. The Family Background Report (FBR) policy in Sri Lanka points to this: in short, the FBR policy restricts Sri Lankan women (mothers) with children below 2 years from migration for paid work based on the gendered argument that the absence of a mother is harmful to a child’s development. In Polanyi’s (2001) perspective, this shows how the state and non-state actors interact and, at times, compete with each other to limit exposure to the market (Goodwin, 2022). However, the logic of the corporation, their business interest, has led brokerage agencies look for their own solutions. A migrant domestic worker in Riyadh, who was not entitled to migrate under the FBR policy, spoke about how her agent undermined state regulations and recruited her for domestic work in Saudi Arabia: ‘The agent knew everything, that I was married and had a child younger than five^6^ years’. Brokerage agencies therefore (re)produce, challenge, negotiate, and alter social inequalities.

The recruitment of migrant workers to the Arabian Gulf does not come cheap. This is particularly the case of Sri Lankan migrant domestic workers, as complained by a Saudi employer: ‘The *agency fee is very high (. . .) We must pay 20,000-25,000 Saudi Riyals [equivalent to* approximately €*4,700‒*€*5,800] for one housemaid. In other sending countries the agency fee is not that high*’. This system of private sponsorship by individual employers called *kafala*^7^ signifies what is perhaps the foremost rule governing the arrangement of migrant domestic labour. *Kafala* creates a form of debt bondage, tying migrant domestic workers’ right to enter, live/work and leave the Arabian Gulf to individual employers. This legal status technically renders migrant domestic workers unfree as it gives employers tremendous power over them (Parreñas, 2021). *Kafala* therefore places them at extreme risk of violence in an exclusively live-in work arrangement.

From this perspective, although migrant domestic workers are free contract partners, they are not independent persons with the right to quit their job freely (Parreñas, 2023). They are forced to surrender their passport and often the mobile phone to the employer immediately upon arrival. These circumstances leave them with no choice but to remain in abusive and exploitative employment situations to maintain and protect their regular migration status and retain the job which they cannot afford to lose. It simply creates conditions of unfreedom or the denial of negative liberty that contradicts the logic of the market. Being trapped is seen as a form of structural violence that leads to labour trafficking and contract-slavery (Parreñas, 2023). In this way, migrant domestic work emulates forced labour. Consequently, migrant domestic workers are subject to various forms of personal violence – physical or psychological, or both and sexual harassment – strategies utilized by employers to maintain their status quo and maximize the labour of their domestic workers (Deshingkar et al., 2019). Unfreedom, violence, and vulnerabilities are therefore inescapable realities in the politics of domestic labour in the Sri Lanka–Gulf care migration regime and the live-in domestic labour arrangement.

On the field level, the Sri Lankan case represents a mode of transnational brokerage marketizing and corporatizing domestic services by interlinking the politics of a labour brokerage state and the *kafala* system. *Kafala* constitutes an autochthonous model of employment, explicitly produced and regulated by the contextual realities in the Gulf, that is, the region-specific cultural factors, social relations, and axes of differentiation that produce everyday life. Brokerage agencies thus create hybrid and unequal arrangements of domestic service in meeting the demands for paid household labour in the receiving countries while enabling greatly desired labour market entry to many Sri Lankan women at the obvious risks of poor terms and conditions of work, violence, and vulnerability. In recruiting an appropriate migrant domestic workforce, they produce the desirability and marketability of migrant domestic workers in terms of bodily form and along lines of gender, age, race (skin colour), nationality, religion, and other forms of difference and inequality while (re)producing hierarchies and layers of subordination in the most intimate sphere.

## A global migration industry in local contexts: Comparing and concluding reflections from the ‘contextual global sociology’ perspective

Domestic work and/or care brokerage is a global phenomenon that constitutes a universal, (trans)nationally, and contextually specified mode of marketizing and corporatizing labour and care in the Global South and North, basing on South–South, South–North, and East–West migrations. On the one hand, brokerage reflects the emergence of new hybrid and unequal modes of service provision and domestic work and home care arrangements, combining the logics of state, market, corporation, family, religion, and profession and new divisions and combinations of paid and unpaid labour with inherent relations of inequality, power and dominance, feminization, ethnicization, and racist and sexist stereotypes. On the other hand, it demonstrates the commodification of domestic labour and care provision in dis/embedded markets in an interplay between the dynamics of the market in terms of demand and supply (middle and upper classes around the globe as clients of labour and care brokerage agencies, primarily female migrant workers from poorer regions or parts of the population). Based on the two case studies, this article thus brings into light the different modes of brokerage in different socio-spatial contexts, where Abraham and Tastsoglou (2016), referring to their contextual global sociological perspective, argue that the realities on the ground for countries and regions vary and they must be framed and understood in relation to specific contexts and connections (e.g. economic, historical, political, cultural, and social). While the contextually embedded modes of brokerage are influenced by the normative and institutional order of the societies involved, they shape the conditions, procedures, and standards in the field which create appropriate migrant workforce in face of clients’ demands. Brokerage is social, cultural, and political as much as it is economic; thus, it should not be perceived as a business operated in disembedded markets, separated from the institutional and normative order of the respective societies (Dammayr, 2019; Findlay et al., 2013; Lee, 2010). Moreover, and coming back to our theoretical entry point, the brokerage business and the marketization and corporatization of the ‘fictitious commodities’ labour and care are not only restricted but also based on their characteristic that they cannot ‘be detached from the rest of life’ (Polanyi, 2001: 75). The recruitment policies and strategies of brokerage agencies cannot be detached from the regional political, social, and cultural contexts (Abraham and Vasil, 2024) in which workforce becomes available and their own business takes shape and can flourish.

The global business of brokerage exploits informality and intimacy of domestic and care work (e.g. private household as a workplace, limited coverage by labour law, informal negotiations between employer/care recipients/relatives and employee/caregivers and poor working conditions). In this way, brokerage offers a critical lens to understanding the context-specific manifestations of inequality, violence, and vulnerability on a global level where migrant domestic and care workers’ strategies for mobility, both geographical and social, are inextricably intertwined with brokerage agents. Brokerage is a constituent element of the contemporary global market capitalism in which delimited exploitation (Gerstenberger, 2018) of migrant workforce in the relations of gender, race, class, and other forms of difference and inequality build the foundation of the business. Therefore, in terms of structural violence, the Austrian and Sri Lankan mode of labour and care brokerage represent the most comparable contexts.

On the contrary, in terms of addressing and mitigating structural and personal violence, the two contexts are the most diverse and far apart, if we compare them on the field level. In the Austrian model as a paradigmatic case of neoliberal free trade of services by intermediating self-employed workers in the frame of a contract triangle (i.e. brokerage agency-household, household-carer, and carer-brokerage agency), the brokerage agencies constitute the most powerful players of the apparently equal contract partners involved. They openly and directly influence the formal regulation of care provision and indirectly and discreetly the working conditions, especially those that are essential for the provision of services. As promoters and lobbyists, they play a role in producing the conditions in the field insofar that they benefit from the informal and poor working conditions and standards without being in charge of them. In this constellation, personal violence becomes an individual problem which seems not to be related to the mode of service provision organized by the brokerage agencies. Nevertheless, personal violence is related to structural violence because the private household is a less protected workplace. Migrant workers recruited as cheap workforce cannot afford to live out even if they feel threatened and being in charge for the care recipient’s health restricts the self-employee’s mobility.

In the Sri Lankan context, characterized by a dual contract model (i.e. separate work contracts between the worker–employer brokerage agency and the worker-employer) in a transnationally regulated market with a level of oversight and control and ostensibly equal contract partners, brokerage agencies act as mediators between the state, employers, and migrant domestic workers. Occupying the middle space, physical (geographical) and institutional distance between employers and migrant domestic workers (Findlay et al., 2013) and combining and contesting the logics of the family, state, market, profession, religion, and so on, and social inequalities, they exercise a greater degree of agency in producing and shaping migrant domestic workers’ spectrum of lived realities experienced in the circuits of transnational migration. While brokerage agencies, as skilled mediators, are integral to migrant domestic workers’ strategies for mobility, they are simultaneously accountable for the (co-)production of exploitative relations and unfree labour through brokerage practices that are intertwined with restrictive im/migration bureaucracies and unequal relations of power in an embedded cross-border labour market. In this way, brokerage is explicitly and implicitly connected with producing inequality, violence, and vulnerability experienced by migrant domestic workers. In the circumstances of institutionalized structural violence, domestic workers are often subject to extreme conditions of personal violence; according to Frantz (2013; cited in Deshingkar et al. (2019)), as candidates of brokerage agencies channelled into state-sponsored bonded labour arrangements.

In this way, the Austrian and Sri Lankan country-studies point to how care and labour brokerage produces new forms and modes of exploitation based on migration regimes. Each commodity form is linked to contextually produced formality and informality, or regularity and irregularity. Viewed from a Polanyian perspective, the commodification of ‘fictitious commodities’ (Polanyi, 2001: 75) in the capitalist market economy thus engages processes of disembedding and (re)embedding. From the institutional logics perspective and intersectional theory, the specific commodity forms and modes interact with the institutional and normative order of the society, the division of labour and the inherent social inequalities. In the contexts of both Austria and Sri Lanka, brokerage has formalized, standardized, and professionalized a service, which is economically and culturally undervalued and poorly socially recognized, both historically and in the twenty-first century. Brokerage agencies have become powerful actors profiting from domestic services, however, often benefitting clients/care recipients/employers at the expense of employees/caregivers/ migrant domestic workers in terms of the conditions and terms of work, thus producing regionally embedded new modes of service provision and forms of care marketization and corporatization, exploitation, and violence.

## Notes

1. Board and lodging is provided in the private household where the domestic or care worker lives and works.

2. Based on Gerstenberger (2018), we focus on the vulnerability of workers. Nevertheless, in the field of domestic service brokerage, children and seniors are also considered as those vulnerable to violence, but this is not the topic of this article.

3. The policy and regime analysis has been conducted as content analysis of all documents which are of relevance to understand how the brokerage business is regulated in Austria, also regarding the regulation of the sending countries and European Union. Eleven expert interviews with stakeholders like leading staff from governmental offices, the Federation of Social Insurances, the Ministry of Social Affairs, Health, Care and Consumer Protection, the Chamber of Labour, the Chamber of Commerce, trade unions, and organizations of the care workers gave further insight into the embeddedness of the brokerage business and the live-in care in Austria. The website analysis covered the advertisement of domestic services presented by all 120 brokerage agencies settled in Vienna. In selected 11 of them, expert interviews with the owners or staff of the brokerage agencies gave insight into their business model regarding the demands, expectations, and claims of all parties involved and the service provision. Three case studies combining non-participatory and participatory observation and episodic interviews were organized as ethnographic in-depth investigation following the brokerage agencies’ activities. The case studies included 26 interviews with all parties involved: the brokerage agencies’ staff, the care recipients, their relatives, the care workers, additional mobile services. Three group discussions covered the field of organizations and initiatives representing the interests of the care workers, the care recipients, and the relatives. Five episodic interviews with already interviewed stakeholders and brokerage agencies and a media analysis covering all reports in all important Austrian newspapers in the first year of the Covid-19 pandemic investigated the respective changes of the business model and the live-in care arrangement.

4. The in-person interviews in Saudi Arabia and Kuwait include 25 interviews with Sri Lankan migrant domestic workers, 20 interviews with employers, 9 interviews with Sri Lankan consular staff, and 5 interviews with brokerage agents that were carried out on the premises of the Sri Lankan embassies; the interviews in Sri Lanka that were conducted digitally include 7 interviews with brokerage agents and 3 interviews each with managerial staff of the Sri Lanka Bureau of Foreign Employment (SLBFE) and non-state agents, including the Association of Licenced Foreign Employment Agents (ALFEA) and the International Organization for Migration (IOM). In addition, a total of five direct personal interviews were carried out by external field assistants in Sri Lanka, including two interviews with brokerage agents and three interviews with managerial staff of the SLBFE. External field assistants were recruited to conduct part of the fieldwork in Sri Lanka due to the political unrest that prevailed in the country at the time, which prevented us from travelling to Sri Lanka. Non-participant field observation in public, semi-public, and private spaces included reception halls, office rooms, markets, restaurants, airports, neighbourhoods, and streets. Secondary data sources, such as policy documents and circulars, provided important insight into the research issue that primary data sources alone could not offer.

5. The rest of the women migrate on self-basis, that is, through private sources, such as with the help of migrant or ex-migrant relatives and friends.

6. The 5-year age limit introduced in 2013 was relaxed and reduced to 2 years in June 2022 to promote labour migration during Sri Lanka’s economic crisis, which is applicable to date.

7. *Kafala* is applied to all categories of temporary foreign labour in the Gulf Cooperation Council (GCC), Jordan, and Lebanon.
